# Emergent Kagome lattice and non-Abelian lattice gauge field of biexcitons in t-MoTe_2_

**DOI:** 10.1093/nsr/nwaf328

**Published:** 2025-08-12

**Authors:** Haochen Wang, Wang Yao

**Affiliations:** New Cornerstone Science Laboratory, Department of Physics, University of Hong Kong, Hong Kong 999077, China; HK Institute of Quantum Science and Technology, University of Hong Kong, Hong Kong 999077, China; New Cornerstone Science Laboratory, Department of Physics, University of Hong Kong, Hong Kong 999077, China; HK Institute of Quantum Science and Technology, University of Hong Kong, Hong Kong 999077, China

**Keywords:** non-Abelian gauge fields, biexciton, twisted bilayer MoTe_2_, genuine non-Abelian AB interference, valley pseudospin qubits

## Abstract

Non-Abelian gauge fields, characterized by their non-commutative symmetry groups, shape physical laws from the Standard Model to emergent topological matter for quantum computation. Here we find that moiré exciton dimers (biexcitons) in the twisted bilayer MoTe$_2$ are governed by a genuine non-Abelian lattice gauge field. These dipolar-bound exciton dimers, formed on bonds of the honeycomb moiré superlattice, exhibit three quadrupole configurations organized into a Kagome lattice geometry, on which the valley-flip biexciton hoppings through electron-hole Coulomb exchange act as link variables of the non-Abelian lattice gauge theory. The emergence of the gauge structure here is a new possibility for composite particles, where the moiré electronic structure and interactions between the electron and hole constituents jointly enforce the underlying geometric constraint. The quadrupole nature of the biexciton further makes possible local gate controls to isolate designated pathways from the extended lattice for exploiting consequences of non-commutative gauge structure, including the genuine non-Abelian Aharonov–Bohm effect. This also provides a new approach for quantum manipulation of the excitonic valley qubit. We show that path interference on a simplest loop can deterministically transform the computational basis states into Bell states.

## Teaser

The authors have uncovered a novel mechanism for the emergence of non-Abelian gauge fields for composite particles, where the electronic structure of its constituents and their interactions jointly enforce the underlying geometric constraint.

## INTRODUCTION

Gauge fields mediate nature’s fundamental forces, governing interactions from subatomic to cosmological scales. In an Abelian gauge field, particles can acquire path-dependent phases that lead to the seminal Aharonov–Bohm (AB) effect. For non-Abelian fields, the behavior is markedly richer: even the order of paths matters due to non-commutativity [1]. Non-Abelian gauge field is central to a list of grand challenges in modern physics, from quantum chromodynamics to topological quantum computing. In artificial quantum systems, gauge fields can emerge through two primary mechanisms: (1) strong many-body correlations (e.g. fractional quantum Hall states) [2] or (2) geometric constraints on single-particle dynamics (e.g. Bloch bands of lattice systems) [1,3]. The latter has been exploited for synthesizing non-Abelian gauge fields in laser-coupled cold-atom systems and optical systems [4–10]. In particular, the non-Abelian AB effect has been demonstrated using fiber optics [9,10], in a one-dimensional ladder of cold atoms [7], and also simulated in electrical circuits [11].

Moiré materials formed by two-dimensional transition metal dichalcogenides (TMDs) have provided a versatile platform for exploring condensed matter frontiers, from quantum matters of fundamental interest [12–22] to excitonic quantum optics and optoelectronic applications [23–29]. TMD moiré systems also make possible engineering emergent gauge fields for electrons, through exploiting the Berry phases arising from the moiré-patterned layer pseudospin texture [30–34]. In twisted MoTe$_2$ moiré superlattices, the Abelian gauge field in the adiabatic dynamics [31] leads to the formation of a flat Chern band in the ferromagnetic phase [17], underpinning the groundbreaking discovery of the fractional quantum anomalous Hall effect at zero magnetic field [18–21]. The non-Abelian gauge field can also emerge from non-adiabatic dynamics in inhomogeneously distorted moiré materials [32], though probing its consequence exceeds existing capabilities. More fundamentally, for all such synthetic gauge field systems, a pivotal question persists: how can these engineered non-Abelian fields be harnessed for practical applications or fundamental advances?

In this work, we uncover a novel mechanism for the emergence of non-Abelian gauge fields in a composite particle, where the electronic structure of its constituents and their interactions jointly enforce the underlying geometric constraint. The particle concerned is a bound dimer of moiré excitons (i.e. a biexciton) in the twisted bilayer MoTe$_2$, formed by excitonic dipole attraction on the nearest-neighbor bonds of the honeycomb moiré superlattice. Such biexcitons are thermodynamically favored over a broad range of intermediate temperatures, and have three quadrupole configurations, in addition to valley pseudospins. We show that electron-hole Coulomb exchange enables valley-flip biexciton hoppings, which organize into a Kagome lattice geometry. These hoppings act as $U(2) \times U(2)$ link variables in a genuine non-Abelian lattice gauge theory, where the two $U(2)$ groups respectively operate on the valley pseudospins of the two individual excitons within a dimer. We find exotic nodal-ring zero modes in the biexciton band dispersion, where the effective mass diverges along high-symmetry lines and vanishes orthogonally. The quadrupole nature of the biexciton further makes possible local gate control to isolate simple pathways from the extended lattice for exploiting the intriguing consequences of the non-commutative gauge structure, including the genuine non-Abelian AB effect. Remarkably, we find that non-Abelian AB interference on simplest loops can realize an entanglement gate for the valley pseudospin qubits, and deterministically transform the computational basis states into Bell states.

**Figure 1.**
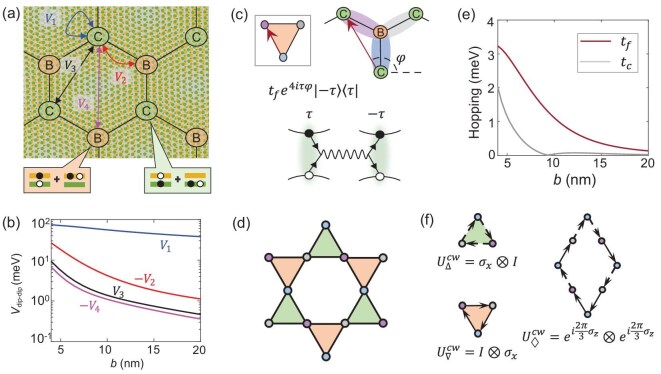
(a) Schematic of a twisted MoTe$_{2}$ moiré superlattice where hybrid excitons are trapped at B and C sites with opposite electric dipoles. (b) Dipole-dipole interactions between hybrid moiré excitons. (c) Biexcitons formed on nearest-neighbor bonds by dipole attraction ($V_{2}$). Red arrow denotes the effective hopping between biexciton configurations, due to the valley-flip electron-hole exchange. Inset shows the equivalent biexciton representation, where color-coded dots denote biexcitons of distinct electric quadrupoles. (d) Emergent Kagome lattice in the biexciton representation. Three biexciton states on each brown (green) triangle share a common B (C) site. (e) Valley-flip ($t_{f}$) versus valley-conserved ($t_{c}$) biexciton hopping as a function of the moiré period *b*. The weight of the intralayer component is taken to be $\frac{1}{6}$ (cf. Note S1). (f) Three loop operators under the dominant valley-flip hopping. Evidently $[U_{\bigtriangledown },U_{\diamondsuit }]\ne 0$, i.e. the lattice gauge field is genuinely non-Abelian.

The twisted MoTe$_2$ bilayer of near 0 degree twisting features interlayer electrical polarization locked to the local stacking registry, which is spatially varying with the moiré periodicity [35,36]. Dipolar interlayer excitons are trapped—by such background electrical polarization—at the MX and XM stacking regions with opposite layer configuration (electric dipole), denoted as sites B and C, respectively, in Fig. 1a. The trapping energy can largely compensate the binding energy difference from an intralayer exciton. This leads to the formation of hybrid moiré excitons at the honeycomb superlattice sites, where the hybridization ratio can be tailored through environmental dielectric engineering that tunes the binding energy difference between inter- and intralayer components. The $C_3$ rotational symmetry at the trapping sites, on the other hand, dictates that the hybridized intralayer component has a *p*-type center-of-mass wave function, where destructive interference leads to a vanishingly small optical transition dipole [37].

**Figure 2.**
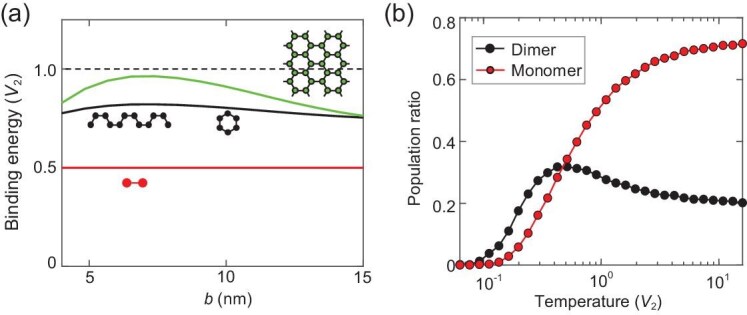
(a) Binding energy per exciton versus the moiré period *b*, in different dipolar bound configurations. Green represents an extended cluster (area$\gg$boundary), black represents an armchair line or hexagon and red represents a dimer (biexciton). (b) Population ratio of moiré excitons that exist in the dimer and monomer forms, respectively, as a function of temperature at a filling of 0.1 excitons per cell. Here $b = 11$ nm, where the energy/temperature unit $V_{2}\sim 3$ meV or 35 K (cf. Fig. 1b). See Note S3 for details of the Monte Carlo simulation.

Such hybrid moiré excitons are thus symmetry protected to have long radiative lifetime, even with a significant intralayer component [37,38]. Through the electric dipole of the interlayer component, they interact repulsively within each sublattice and attractively between the two sublattices. Figure 1b plots the strength of four leading interaction channels as function of the moiré period *b* (cf. Note S2). On-site repulsion $V_{1}$ of the order of tens of millielectronvolts effectively excludes double occupation at low exciton temperature. The dominant interaction channel becomes the nearest-neighbor (NN) attraction $V_{2}$, which binds excitons into dimers, or a biexciton that carries electric quadrupole moment on the NN bond. Such biexcitons have a sizable binding energy $\sim\! 10$ meV at a moiré period of $\sim\! 10$ nm (Fig. 1b). Excitons can further segregate into larger clusters, which are also affected by the much weaker next-NN repulsion $V_3$ and next-next-NN attraction $V_4$, and so on (cf. Note S3). The additional energy gain by forming larger clusters is not as significant as that upon forming dimers, as shown by plots of the binding energy per exciton in Fig. 2a. This means that, at finite temperature where the exciton clusters dissociate by thermal fluctuations, biexcitons can be favored. Figure 2b shows the ratio of moiré hybrid excitons that exist in the dimer form at a filling of 0.1 excitons per cell, from Monte Carlo simulations. The biexciton ratio exceeds $30\%$ at 20 K, and remains significant up to room temperature. We focus on such quadrupolar biexcitons, which are of clear experimental relevance in a thermal gas over a broad range of intermediate temperatures, and in the dilute limit down to low temperature.

## BIEXCITON HOPPING ON THE EFFECTIVE KAGOME LATTICE

These biexcitons exhibit three distinct quadrupole configurations determined by the orientation of NN bonds, as highlighted by the oval shades in Fig. 1c. Two biexciton configurations sharing a common B or C site can be interconverted through a single-step hopping of one exciton in the dimer. This enables the biexciton to hop in the moiré superlattice as easily as an individual exciton, whereas the spatial configurations and their connections constitute an effective Kagome lattice, with three orbitals of distinct quadrupoles denoted as $D_{1}$, $D_{2}$ and $D_{3}$. Each orbital also has an internal degree of freedom spanned by $\lbrace |\tau \rangle _{B}|\tau ^{\prime }\rangle _{C}$}, where $\tau ,\tau ^{\prime }=\pm$ are the excitonic valley pseudospins at sites B and C, optically accessible through the valley selection rule [38]. Note that this biexciton Kagome lattice only has nearest-neighbor hopping: any direct further-neighbor hopping is a second-order process through a far-detuned two-exciton configuration, and therefore negligibly small.

Generally, hopping of hybrid excitons in the moiré superlattice takes place through two microscopic mechanisms [37]: (i) the valley-conserving kinetic propagation in the moiré potential [39]; (ii) the electron-hole Coulomb exchange, which can annihilate a $\tau$-valley exciton at one moiré site and non-locally create one in valley $\tau ^{\prime }$ at another site (cf. Fig. 1c). The latter is essentially the Förster coupling, well known as an energy transfer mechanism [40–44]. Here it plays the role of a coherent hopping channel between the sites of the moiré superlattice, which is activated only when the exciton has an intralayer component. Such a Förster coupling channel for the hybrid moiré excitons in twisted MoTe$_2$ has been systematically analyzed in [37]. For the biexcitons here, the relevant channels are the valley-flip and valley-conserved hoppings between a closest pair of sites both on the B or C sublattice (cf. Fig. 1c). In Fig. 1e, we compare the valley-flip and valley-conserved hopping strengths as functions of the moiré period. Notably, the valley-flip Förster coupling has a longer range compared to the valley-conserved one, with the latter further subject to destructive interference with the kinetic propagation (see Note S1). As a result, the valley-flip process dominates the biexciton hopping on the Kagome lattice, which takes the form $t_f (e^{4i \varphi } | - \rangle \langle + | + e^{-4i \tau \varphi } | + \rangle \langle - |)$ with $\varphi$ the angle of the hopping direction.

## NON-ABELIAN LATTICE GAUGE FIELD

Biexcitons in t-MoTe$_2$ are therefore described by the low-energy effective Hamiltonian


(1) \begin{eqnarray*}
\widehat{H}&=\sum _{\vec{r}}t_{f}(\hat{b}^{\dagger }_{1,\vec{r}}\,\hat{h}_{1}\hat{b}_{2,\vec{r}} +\hat{b}^{\dagger }_{2,\vec{r}}\,\hat{h}_{2}\hat{b}_{3,\vec{r}}+\hat{b}^{\dagger }_{3,\vec{r}} \,\hat{h}_{3}\hat{b}_{1,\vec{r}})\\
&\quad +\sum _{\vec{r}}t_{f}(\hat{b}^{\dagger }_{1,\vec{r}}\,\hat{h}^{\prime }_{1}\hat{b}_{2,\vec{r} +\vec{\delta _{1}}}+\hat{b}^{\dagger }_{2,\vec{r}}\,\hat{h}^{\prime }_{2}\hat{b}_{3,\vec{r} +\vec{\delta _{2}}}\\
&+\hat{b}^{\dagger }_{3,\vec{r}}\,\hat{h}^{\prime }_{3}\hat{b}_{1,\vec{r}+\vec{\delta _{3}}})+{\rm H.c.}
\end{eqnarray*}

Here $\hat{b}^{\dagger }_{1,\vec{r}}$, $\hat{b}^{\dagger }_{2,\vec{r}}$ and $\hat{b}^{\dagger }_{3,\vec{r}}$ are the creation operators of the $D_{1}$, $D_{2}$ and $D_{3}$ biexcitons at the Kagome unit cell centered at $\vec{r}$; the $\vec{\delta }_{i}$ are primitive lattice vectors, related by $C_{3}$ rotation; $\hat{h}_{j} \equiv \hat{I}\otimes (\hat{\sigma }_{x} \cos \varphi _{j} + \hat{\sigma }_{y} \sin \varphi _{j})$ and $\hat{h}^{\prime }_{j} \equiv ( \hat{\sigma }_{x} \cos \varphi _{j} + \hat{\sigma }_{y} \sin \varphi _{j})\otimes \hat{I}$ are the valley-flip operators upon the biexciton hopping, respectively acting on the dipole-up exciton (C site) and dipole-down exciton (B site); $\varphi _{j}=0,{2\pi }/{3},-{2\pi }/{3}$ are the angles of hopping directions $D_{1}\rightarrow D_{2}$, $D_{2}\rightarrow D_{3}$ and $D_{3}\rightarrow D_{1}$, respectively. Valley-conserved hopping is neglected here because of its small magnitude, especially for moiré period $b \ge 10$ nm (cf. Fig. 1e).

When $\varphi _j\ne \varphi _{j^{\prime }}$, $[\hat{h}_{j},\hat{h}_{j^{\prime }}]\ne 0$ and $[\hat{h}^{\prime }_{j}, \hat{h}^{\prime }_{j^{\prime }}]\ne 0$, namely, the valley rotations upon the hoppings are non-commutative. Hoping $\hat{h}_{j}$ and $\hat{h}^{\prime }_j$ are the elements of a $U(2)\times U(2)$ group, and the hopping processes in Equation (1) act as link variables for a corresponding non-Abelian lattice gauge theory. The non-Abelian properties are illustrated by three simplest loop paths in Fig. 1f. The loop operators are given by $U^{CW}_{\bigtriangleup }=\hat{\sigma }_{x}\otimes I$, $U^{CW}_{\bigtriangledown }=I\otimes \hat{\sigma }_{x}$ and $U^{CW}_{\diamondsuit }=e^{{2 i\pi } \hat{\sigma }_{z} /{3}}\otimes e^{{2 i\pi }\hat{\sigma }_{z}/ {3} }$, which realize valley flips of dipole-down and dipole-up excitons, and the valley-dependent phase shift, respectively. The non-commutativity $[U_{\bigtriangledown },U_{\diamondsuit }]\ne 0$ characterizes the genuine non-Abelian nature of this $U(2)\times U(2)$ lattice gauge field [1].

## BIEXCITON DISPERSION

**Figure 3.**
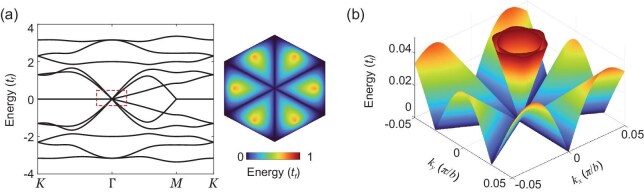
(a) Biexciton bands in the Kagome lattice, featuring doubly degenerate nodal-ring zero modes along the high-symmetry lines $\bar{K}$-$\Gamma$-*K* and $\bar{K}$-*M*-*K*. Energy is in units of $t_{f}$, the valley-flip hopping amplitude. (b) Dispersion near the $\Gamma$ point (cf. dashed box in (a)).

Figure 3 presents the dispersion of biexcitons under the non-Abelian lattice gauge field in Equation (1). There exist doubly degenerate nodal-ring zero modes, where the biexciton effective mass diverges along the high-symmetry lines $\bar{K}$-$\Gamma$-*K* and $\bar{K}$-*M*-*K*, and vanishes orthogonally with a Dirac-like dispersion. The zero lines cross at the $\Gamma$ point with a six-fold degeneracy, where two additional Dirac cones emerge (Fig. 3b). The gauge field here preserves three symmetries: (i) time-reversal symmetry; (ii) particle-hole symmetry and (iii) chiral symmetry. The $U(2)\times U(2)$ lattice gauge structure underlies the form of the Hamiltonian having off-diagonal blocks only, and the rank of the off-diagonal block determines the existence and degeneracy of nodal-ring zero modes along high-symmetry lines [45]. See Note S4 for details.

## NON-ABELIAN AB EFFECT AND BELL STATE PRODUCTION UNDER LOCAL GATE CONTROL

**Figure 4.**
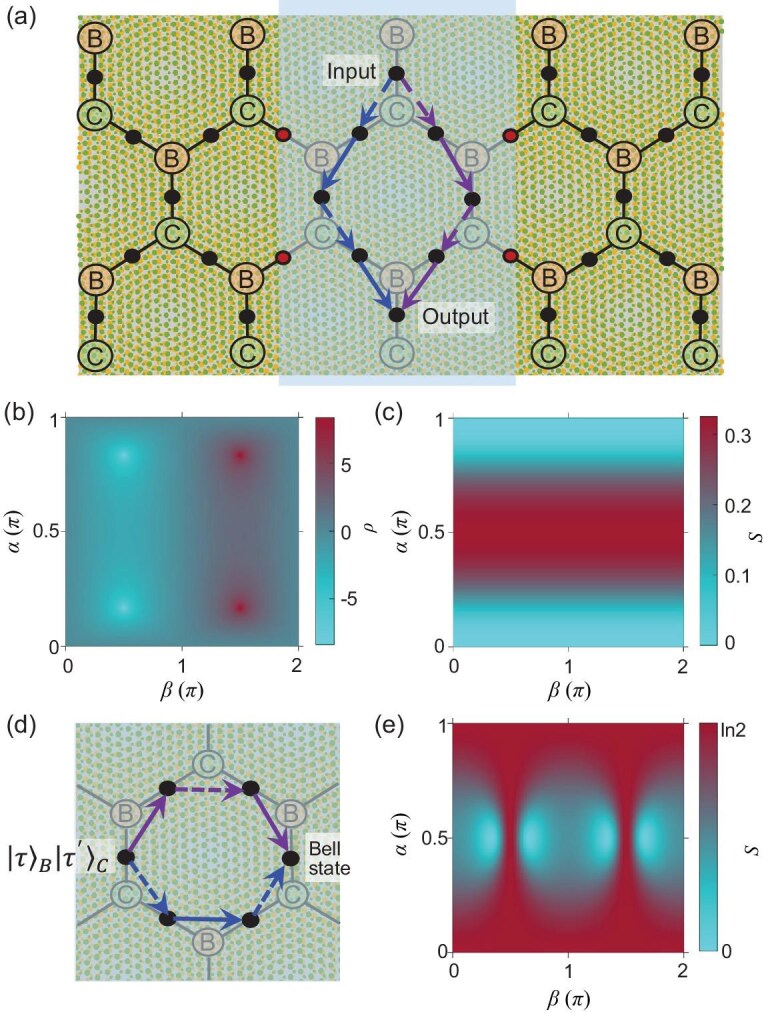
(a) Local gate control for effective isolation of pathways for the non-Abelian Aharonov–Bohm effect. Because of the quadrupole moment of the biexciton, a Stark shift by the interlayer bias occurs only at the boundary of the gated area (red dots). By such detuning, biexciton lattice sites inside can be decoupled from outside. (b and c) Non-Abelian AB interference of evolutions along the two symmetric pathways in (a) with initial state $|\vec{n}\,\rangle _{B}|\vec{n}\,\rangle _{C}$. We plot $\rho =\log |{\langle \leftarrow \leftarrow |\Psi _{f}\rangle }/{\langle \rightarrow \rightarrow |\Psi _{f}\rangle }|^{2}$ and the entanglement entropy *S* in (b) and (c), respectively, for the path-interfered final state, as a function of the Bloch sphere angles of the initial state $|\vec{n}\rangle =[\cos ({\alpha }/{2}),\sin ({\alpha }/{2})e^{i\beta }]^{T}$. (d) Non-Abelian AB interference around a hexagon turns an initial state $|\tau \rangle _{B}|\tau ^{\prime }\rangle _{C}$ into the pure Bell state $e^{i\tau ^{\prime }{2\pi }/{3}}|-\tau \rangle _{B}|\tau ^{\prime }\rangle _{C}+ e^{-i\tau {2\pi }/{3}}|\tau \rangle _{B}|-\tau ^{\prime }\rangle _{C}$. Two Bell states can therefore be deterministically realized. (e) Entanglement entropy of the final state upon the path interference in (d), for the same sets of initial states as in (b) and (c).

The moiré period $b \ge 10$ nm is the favorable parameter regime for exploring the non-Abelian lattice gauge field and the Bell state generation, where the valley-conserved hopping vanishes whereas the valley-flip hopping still has a significant magnitude. Electric gate control of the biexciton is made possible through its quadrupole moment, which can couple to the lateral gradient of a perpendicular electric field. Such a field gradient can be naturally realized at the boundary of a top or bottom gate, as schematically illustrated in Fig. 4a. On honeycomb bonds either inside or outside the gated area, the biexciton energy does not respond to the interlayer bias, as Stark shifts of its B and C components cancel each other. On partially covered bonds at the boundary (cf. red dots in Fig. 4a), the biexciton energy can acquire a Stark shift of the order of the interlayer bias. Either a positive or negative energy shift $\gg t_f$ effectively decouples the affected lattice sites. A modest local gate bias can therefore be implemented for isolating the designated area from the rest of the lattice.

The gated area illustrated in Fig. 4a encloses a diamond-shaped interference pathway, for which we examine the AB effect as well as valley pseudospin manipulations in the non-Abelian lattice gauge field. For biexcitons in a valley basis state $|\tau \rangle _{B}|\tau ^{\prime }\rangle _{C}$, the evolution along the blue and purple pathways with an even number of valley flips will end up in the same state $|\tau \rangle _{B}|\tau ^{\prime }\rangle _{C}$ with phase changes of $e^{i(\tau + \tau ^{\prime }){2\pi }/{3}}$ and $e^{-i(\tau + \tau ^{\prime }){2\pi }/{3}}$, respectively. The $\tau , \tau ^{\prime }$ dependence of the phase leads to path-dependent valley rotations about the *z* axis for a general initial state, which is characteristic of the non-Abelian AB effect [1,7,46]. Consider an initial state $|\vec{n}\rangle _{B}|\vec{n}\rangle _{C}$, where $\vec{n}$ is a general valley Bloch vector common for both B and C components. Along either the blue or purple path, the propagation in the gauge field preserves this form of wave function, leading to a common valley rotation for B and C components with path dependence. This results in a final state of the form $|\Psi _{f}\rangle = e^{-i{2\pi }/{3}}|\vec{n}_1\rangle _{B}|\vec{n}_1\rangle _{C} + e^{i{2\pi }/{3}}|\vec{n}_2\rangle _{B}|\vec{n}_2\rangle _{C}$. The final state is characterized in panels (b) and (c) of Fig. 4 in terms of the valley polarization ratio $\rho =\log |{\langle \leftarrow \leftarrow |\Psi _{f}\rangle }/{\langle \rightarrow \rightarrow |\Psi _{f}\rangle }|^{2}$ and entanglement entropy *S*, as a function of the sphere angles of the initial Bloch vector $\vec{n}$. Characteristics of the non-Abelian AB effect are clearly seen here [7,46]. Through the valley optical selection rule, the valley polarization ratio $\rho$ can manifest in the linear polarization angles of the photons from biexciton emission.

The non-Abelian AB interference in the local-gate-defined pathway can therefore be implemented for entanglement generation between the valley pseudospins of the exciton components, which can serve as qubit carriers given their long radiative lifetime. In Fig. 4d, we exploit the interference pathway on a single hexagon unit, for generation of maximum entanglement. Consider an input on any of the basis states $|\tau \rangle _{B}|\tau ^{\prime }\rangle _{C}$; upon propagation along the blue and purple paths, the final state of the interference becomes $e^{i\tau ^{\prime }{2\pi }/{3}}|-\tau \rangle _{B}|\tau ^{\prime }\rangle _{C}+e^{-i\tau {2\pi }/{3}}|\tau \rangle _{B}|-\tau ^{\prime }\rangle _{C}$, which is always a Bell state. Figure 4e plots the entanglement entropy of the final state upon the path interference for a general initial state $|\vec{n}\rangle _{B}|\vec{n}\rangle _{C}$, where maximal entanglement ($S=\ln 2$) is achieved over a broad region of the initial state parameter space. This showcases a new form of quantum controls in a non-Abelian lattice gauge field.

Lastly, we note that such biexcitons are not limited to twisted MoTe$_2$ bilayers, but may be generally expected in near 0 degree twisting TMD homobilayers, as long as *K*-valley excitons are concerned. However, significant quantitative differences need to be noted. In TMD compounds other than MoTe$_2$, *K*-valley excitons may not be the lowest-energy configurations in homobilayers, and therefore subject to relaxation to other momentum indirect excitons. Besides, the quantitative difference of the background electrical polarization in other twisted TMDC compounds can lead to different energy detunings between interlayer and intralayer excitons, and therefore a different hybridization ratio, which determines the strength of the Förster coupling.

## Supplementary Material

nwaf328_Supplemental_File
