# Supplementary material for: Emergent Kagome lattice and non-Abelian lattice gauge field of biexcitons in t-MoTe2
Source: Natl Sci Rev. 2025 Aug 12;13(4):nwaf328. doi: 10.1093/nsr/nwaf328 (PMC12878358; doi:10.1093/nsr/nwaf328)
Supplement: nwaf328_Supplemental_File [file nwaf328_supplemental_file.pdf]

Supplementary Material for
"Emergent Kagome lattice and non-Abelian lattice gauge field of biexcitons in t-$MoTe_2$"

Haochen Wang[1,2] and Wang Yao[1,2*]
[1] *New Cornerstone Science Laboratory, Department of Physics, University of Hong Kong, Hong Kong, China and*
[2] *HK Institute of Quantum Science and Technology, University of Hong Kong, Hong Kong, China*

Supplementary Note 1. Hybrid moiré exciton wavepackets and relevant hopping matrix elements in t-$MoTe_2$

We consider the wavefunction forms of hybrid moiré excitons trapped at B and C high symmetry points and their hopping matrix elements between NNN moiré trapping sites that are relevant for biexcitons (c.f. Fig. 1c in main text). We use $\psi^{\mu\nu}_{m,\tau}$ to describe an exciton from τK valley, with $\mu = c, c'$ and $\nu = v, v'$ being the layer index of electron and hole constituents, respectively (the prime notation denoting bottom layer). $W_m(\boldsymbol{R})$ denotes the center-of-mass (COM) part of $\psi^{\mu\nu}_{m,\tau}$, characterized by the azimuthal quantum number m, $\boldsymbol{R}$ being exciton's COM coordinates [1]. At high-symmetry points, they are eigenstates of the threefold rotational operator $\hat{C}_3$, satisfying: $\hat{C}_3 W_m(\boldsymbol{R}) = W_m(\hat{C}_3^{-1}\boldsymbol{R}) = e^{-i\frac{2\pi}{3}m} W_m(\boldsymbol{R})$. The hybridization between interlayer and intralayer excitons is determined by the interlayer tunneling matrix T between valence or conduction band edges of $MoTe_2$ at the τK valley. In R-stacking bilayers, the stacking selection rule requires the tunneling matrix element T to vanish at the B and C high-symmetry points [1–3]. In their vicinity, the phase of T matrix element exhibits vortex configurations with winding number τ and $-\tau$ at B and C points, respectively [4]. Considering the dominant valence band hopping [4], the hybridization matrix element should satisfy the rotational symmetry:

$$\langle \psi^{cv}_{m,\tau} | \hat{T} | \psi^{cv'}_{0,\tau} \rangle = \langle W_m | T_{vv',\tau}(\boldsymbol{R}_B) | W_0 \rangle = \langle \hat{C}_3 W_m | \hat{C}_3 T_{vv',\tau}(\boldsymbol{R}_B) \hat{C}_3^\dagger | \hat{C}_3 W_0 \rangle = e^{i\frac{2\pi}{3}(m-\tau)} \langle \psi^{cv}_{m,\tau} | \hat{T} | \psi^{cv'}_{0,\tau} \rangle \,, \tag{S1}$$

$$\langle \psi^{c'v'}_{m,\tau} | \hat{T} | \psi^{c'v}_{0,\tau} \rangle = \langle W_m | T^*_{vv',\tau}(\boldsymbol{R}_C) | W_0 \rangle = \langle \hat{C}_3 W_m | \hat{C}_3 T^*_{vv',\tau}(\boldsymbol{R}_C) \hat{C}_3^\dagger | \hat{C}_3 W_0 \rangle = e^{i\frac{2\pi}{3}(m-\tau)} \langle \psi^{c'v'}_{m,\tau} | \hat{T} | \psi^{c'v}_{0,\tau} \rangle \,. \tag{S2}$$

We consider the situation of interlayer component always having a s-type COM envelope due to the strong moiré trapping. The above hybridization matrix element then requires the hybridized intralayer component having a p_+ (p_-) type COM envelope at K (-K) valley, at both B and C sites [3, 5].

The hybrid moiré exciton can be generally written as follows:

$$\begin{aligned} |\Psi^{\rm hy}_{B,\tau}\rangle &= e^{-i\theta\tau/2}(\mathcal{W}_0 |\psi^{cv'}_{\tau,0}(\boldsymbol{R}_B)\rangle + \mathcal{W}_\tau |\psi^{cv}_{\tau,t,\tau}(\boldsymbol{R}_B)\rangle), \\ |\Psi^{\rm hy}_{C,\tau}\rangle &= e^{i\theta\tau/2}(\mathcal{W}_0 |\psi^{c'v}_{\tau,0}(\boldsymbol{R}_C)\rangle + \mathcal{W}_\tau |\psi^{c'v'}_{\tau,b,\tau}(\boldsymbol{R}_C)\rangle), \end{aligned} \tag{S3}$$

Here, we have followed the convention of Ref. [5] for the overall phase factor, and also take the weight of the intralayer component $|\mathcal{W}_\tau|^2 = \frac{1}{6}$. There can be two contributions to the effective hopping of such hybrid moiré excitons between NNN superlattice sites (i.e. between a closest pair of sites on the same sublattice). The first is kinetic propagation, dominated by the primary interlayer component. Under the present gauge choice, it is of the form $t_k = e^{\frac{2\pi}{3}\tau} t$, where the phase factor is due to the momentum space mismatch between the τK points of the two layers [5, 6], and the amplitude t is exponentially decaying as the function of distance (Supplementary Figure 1b). The second contribution to the NNN hopping is by the Förster coupling through the intralayer component. For the p-type COM envelope, the valley-flip and valley-conserved Förster coupling channels have been systematically analyzied in Ref. [5]. Their weighted contribution to the hybrid exciton hopping matrix element are:

$$\begin{aligned} \langle \Psi^{\rm hy}_{-\tau} | \hat{J} | \Psi^{\rm hy}_{\tau} \rangle &= -|\mathcal{W}_\tau|^2 \langle \psi^{\rm intra}_{-\tau} | \hat{J} | \psi^{\rm intra}_{\tau} \rangle = e^{4i\tau\varphi} |\mathcal{W}_\tau|^2 \mathfrak{J}^{-\tau,-\tau}_{\tau,\tau}, \\ \langle \Psi^{\rm hy}_{\tau} | \hat{J} | \Psi^{\rm hy}_{\tau} \rangle &= |\mathcal{W}_\tau|^2 \langle \psi^{\rm intra}_{\tau} | \hat{J} | \psi^{\rm intra}_{\tau} \rangle = |\mathcal{W}_\tau|^2 \mathfrak{J}^{\tau,\tau}_{\tau,\tau}, \end{aligned} \tag{S4}$$

The coupling strength $\mathfrak{J}^{-\tau,-\tau}_{\tau,\tau}$ and $\mathfrak{J}^{\tau,\tau}_{\tau,\tau}$ as functions of moiré period b (NNN distance) are shown in Supplementary Figure 1a.

* wangyao@hku.hk

The valley-conserved NNN hopping is therefore the interference between the Förster coupling and kinetic propagation: $t_c e^{i\tau\nu} = e^{i\frac{2\pi}{3}\tau} t + |\mathcal{W}_\tau|^2 \mathfrak{J}^{\tau,\tau}_{\tau,\tau}$. And the valley-flip NNN hopping is sorely from Förster coupling, which has the from $t_f e^{4i\tau\varphi}$ with amplitude $t_f = |\mathcal{W}_\tau|^2 \mathfrak{J}^{-\tau,-\tau}_{\tau,\tau}$ plotted in Fig. 1e of the main text. φ here is the angle of the hopping direction.

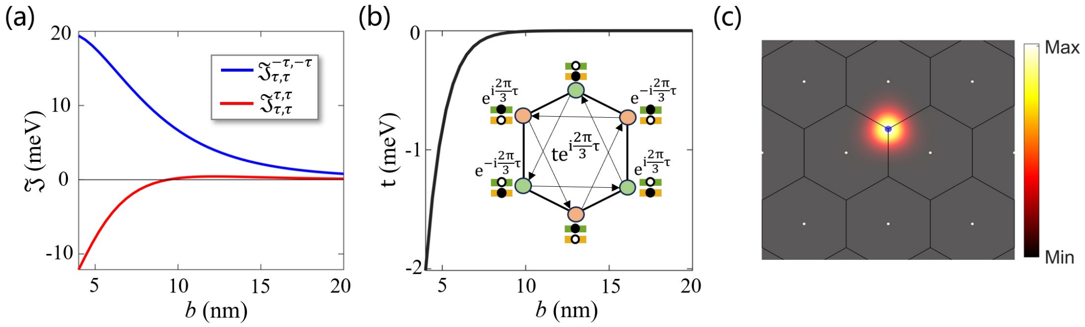

Supplementary Figure 1. (a) The Förster coupling strength of two different channels as functions of moiré period b. (b) the kinetic propagation amplitude as a function of moiré period b, and τ is the valley index. (c) The Wannier function of interlayer exciton at C point.

Supplementary Note 2. Dipole-dipole interaction between two interlayer excitons

In this section, we determine the maximally localized Wannier function of the interlayer exciton, and then use it to calculate the dipole-dipole interaction strengths at different moiré period b.

The moiré potentials of excitons are given as follows [3].

$$\begin{aligned} E_{ij}(\boldsymbol{R}) &= E_i(\boldsymbol{R}) - E_j(\boldsymbol{R}), \\ E_c(\boldsymbol{R}) &= -\delta_c[f_+ + f_-] + \Delta_c[f_+ - f_-], \\ E_{c'}(\boldsymbol{R}) &= -\delta_c[f_+ + f_-] - \Delta_c[f_+ - f_-], \\ E_v(\boldsymbol{R}) &= -\delta_v[f_+ + f_-] + \Delta_v[f_+ - f_-], \\ E_{v'}(\boldsymbol{R}) &= -\delta_v[f_+ + f_-] - \Delta_v[f_+ - f_-]. \end{aligned} \tag{S5}$$

Here, $f_\pm = \frac{1}{9}|e^{i\delta\boldsymbol{K}\cdot\boldsymbol{R}} + e^{i(\hat{C}_3\delta\boldsymbol{K}\cdot\boldsymbol{R}\pm 2\pi/3)} + e^{i(\hat{C}_3^2\delta\boldsymbol{K}\cdot\boldsymbol{R}\pm 4\pi/3)}|$ are the lowest harmonic approximation with rotational and translational symmetry of local stacking registry [4]. For twisted bilayer $MoTe_2$, density functional theory gives these parameters: $\Delta_c = 31\ meV$, $\Delta_v = 42\ meV$, $\delta_c = 2\ meV$, and $\delta_v = 0.5\ meV$ [7]. In terms of two type of interlayer excitons, their envelope wavefunctions are described by the following wave equations:

$$\begin{aligned} i\hbar\frac{W^{cv'}}{\partial t} &= [-\frac{\hbar^2\nabla^2_{\boldsymbol{R}}}{2M} + E_{cv'}(\boldsymbol{R})]W^{cv'}, \\ i\hbar\frac{W^{vc'}}{\partial t} &= [-\frac{\hbar^2\nabla^2_{\boldsymbol{R}}}{2M} + E_{vc'}(\boldsymbol{R})]W^{vc'}, \end{aligned} \tag{S6}$$

Here, $M = m_e$ (m_e is the electron mass). We firstly diagonalize Eq. S6 to get the Bloch states of the lowest energy branch, and then minimize the localization functional [8] to find out a gauge for the maximally localized Wannier function of interlayer excitons. Due to inversion symmetry, the maximally localized Wannier functions of lowest energy branch satisfy $W_0^{cv'}(\boldsymbol{R}) = W_0^{vc'}(-\boldsymbol{R})$ and the former one is shown in Supplementary Figure 1c.

The screened Coulomb interaction in homobilayers is given by Keldysh form: $V(r) = -\frac{e^2}{2\epsilon r_0}[ln(\frac{r}{r+r_0}) + (0.5772 - ln2)e^{-\frac{r}{r_0}}]$ [9, 10]. Here, r_0 is the effective screening length of t-$MoTe_2$, and ϵ is average dielectric constant of an environment where the t-$MoTe_2$ is embedded. Here we use $\epsilon = 3.9\epsilon_0$ with ϵ_0 being the vacuum dielectric constant [9–11]. The dipolar interaction between two hybrid moiré excitons is then given by: $U(\boldsymbol{r}) = 2(V(\boldsymbol{r}) - V(\boldsymbol{r}+\boldsymbol{z}))$, where $\boldsymbol{r}$ is the lateral displacement vector, and $\boldsymbol{z}$ is the displacement vector along z-axis between the two layers. Here we select $z = 0.7$ nm. The dipole-dipole interaction strength between the hybrid moiré exciton wavepackets is then:

$$\begin{aligned} V_i &= (-1)^i \langle \Psi^{\rm hy}(\boldsymbol{R_i})\Psi^{\rm hy}(\boldsymbol{R_C})|U(\boldsymbol{R_i} - \boldsymbol{R_C})|\Psi^{\rm hy}(\boldsymbol{R_i})\Psi^{\rm hy}(\boldsymbol{R_C})\rangle \\ &= (-1)^i |\mathcal{W}_0|^4 \langle W_0^{\rm inter}(\boldsymbol{R_i})W_0^{\rm inter}(\boldsymbol{R_C})|U(\boldsymbol{R_i} - \boldsymbol{R_C})|W_0^{\rm inter}(\boldsymbol{R_i})W_0^{\rm inter}(\boldsymbol{R_C})\rangle, \end{aligned} \tag{S7}$$

Here, $\boldsymbol{R_C}$ is the center of mass location of interlayer exciton at high-symmetry point C, and $\boldsymbol{R_i}$ is at the ith-order neighbor of $\boldsymbol{R_C}$. W_0^{inter} is the maximally localized Wannier function as obtained above. By numerically integrating Eq. S7, the four dipole-dipole interaction channels are plotted in the main text.

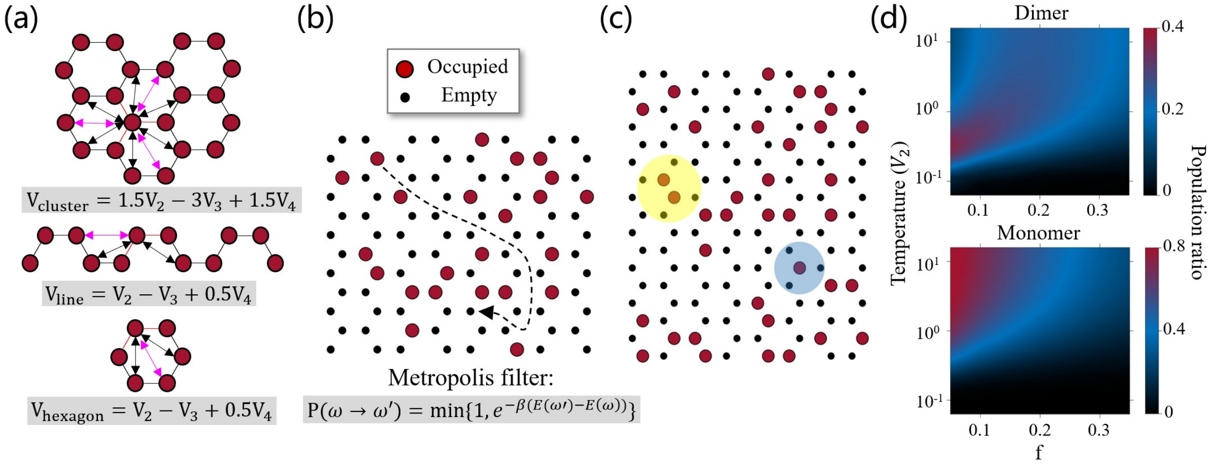

Supplementary Figure 2. (a) The binding energy per exciton of three bound states (b) Metropolis filter of the update scheme. (c) The counting method of dimer (yellow region) and monomer (blue region). (d) The population ratio of dimer (upper) and monomer (bottom) are plotted as the function of temperature and filling factor f.

Supplementary Note 3. Binding energy and Monte Carlo simulations

At zero temperature, all of excitons become localized, forming either cluster, armchair line, or hexagon as a result of the competition between attractive interaction and repulsive interaction. These dipolar bound states are characterized by the binding energy per exciton $E_{cluster} = 1.5V_2 - 3V_3 + 1.5V_4$ and $E_{line} = E_{hexagon} = V_2 - V_3 + 0.5V_4$ as illustrated in Supplementary Figure 2a. The ground state is the configuration of the largest binding energy per exciton. As shown in the main text, at large twist angles, the excitons are bounded as a cluster-ordered state, whereas at small twist angles, the cluster segregate into either armchair lines or hexagons which have same binding energy. As for the cluster, the shape is determined by minimizing the length of boundary to minimize total energy.

At finite temperatures, the Monte Carlo algorithm is applied to calculate the thermal properties of exciton [12]. To maintain fixed filling, the configuration of excitons $\omega = \{n_i\}$ in the lattice is updated into another configuration $\omega' = \{n_i'\}$ via transferring one exciton to a vacant site, as schematically illustrated by the dashed black arrow in Supplementary Figure 2b. The acceptance probability of the update is given by Metropolis filter $P(\omega \to \omega') = min\{1, \frac{e^{-\beta E(\omega')}Q(\omega' \to \omega)}{e^{-\beta E(\omega)}Q(\omega \to \omega')}\}$, where $Q(\omega' \to \omega)$ is the Markov chain transition matrix and our update scheme satisfy detailed balance $Q(\omega' \to \omega) = Q(\omega \to \omega')$. Finally, the acceptance probability is simplified as

$$P(\omega \to \omega') = min\{1, e^{-\beta E(\omega') + \beta E(\omega)}\} \tag{S8}$$

In our simulations, the initial configuration $\{n_i\}$ of excitons is given randomly, and then more than 60000 Monte Carlo step is set to prepare the system. Due to the strong interaction strength, we set all the hopping equal zero. We consider finite-length toroidal with $N_x \times N_y$ sites and total lattice sites $N = N_x \times N_y$. Here, $N_x = 40$ and $N_y = 60$, and the filling is set as $f = 1/10$ in the main text. Thus, the number of excitons is $N_e = fN = 240$.

Besides, more than 60000 Monte Carlo steps are applied to statistically count dimer and monomer after the system is prepared well. A single dimer and a single monomer are defined by the yellow circle and the blue circle as shown in Supplementary Figure 2c, respectively. All the neighbors of them are empty. The population of dimer and monomer are normalized by exciton filling number N_e as shown in Supplementary Figure 2d. With the number of excitons increase, due to the limit lattice site, the probability of two exciton encountering increases, which leads to the dimer and monomer population decrease and the transition temperature increase.

Supplementary Note 4. Symmetry analysis and nodal-ring zero modes

The tight binding Hamiltonian can be written in the momentum space by making the Fourier transformation. $\hat{b}_{\boldsymbol{k}} = \frac{1}{\sqrt{N}} \sum_{\vec{r}} [\hat{b}_{1,\vec{r}}, \hat{b}_{2,\vec{r}}, \hat{b}_{3,\vec{r}}]^T e^{i\boldsymbol{k}\cdot\vec{r}}$, where $\hat{b}^\dagger_{1,\vec{r}}$, $\hat{b}^\dagger_{2,\vec{r}}$, and $\hat{b}^\dagger_{3,\vec{r}}$ are the creation operator of the D_1, D_2, and D_3 biexciton at the Kagome unit cell centered at $\vec{r}$. N denotes the number of cells in total. The kernel Hamiltonian of biexciton in momentum space is then written as

$$H_{\boldsymbol{k}} = t_f \begin{pmatrix} 0 & h_1 + h_1' e^{i\boldsymbol{k}\cdot\boldsymbol{\delta}_1} & h_3 + h_3' e^{-i\boldsymbol{k}\cdot\boldsymbol{\delta}_3} \\ h_1 + h_1' e^{-i\boldsymbol{k}\cdot\boldsymbol{\delta}_1} & 0 & h_2 + h_2' e^{i\boldsymbol{k}\cdot\boldsymbol{\delta}_2} \\ h_3 + h_3' e^{i\boldsymbol{k}\cdot\boldsymbol{\delta}_3} & h_2 + h_2' e^{-i\boldsymbol{k}\cdot\boldsymbol{\delta}_2} & 0 \end{pmatrix} \tag{S9}$$

1. Under time reversal (TR) transformation, both of the valley pseudospin of the dipole up (C site) and dipole down (B site) excitons will flip while the sublattice freedom is kept invariant, so the representation is $\hat{\mathcal{T}} = (I \otimes \sigma_x \otimes \sigma_x)\mathcal{K}$, where $\mathcal{K}$ is complex conjugate (where I is a 3×3 identity matrix).

$$\hat{\mathcal{T}} H_{\boldsymbol{k}} \hat{\mathcal{T}}^{-1} = (I \otimes \sigma_x \otimes \sigma_x) H^*_{\boldsymbol{k}} (I \otimes \sigma_x \otimes \sigma_x)^{-1} = H_{-\boldsymbol{k}}, \tag{S10}$$

The Hamiltonian is TR invariant.

2. Under the particle-hole transformation $\hat{\mathcal{T}}_p = (I \otimes \sigma_y \otimes \sigma_y)\mathcal{K}$, the Hamiltonian has $\hat{\mathcal{T}}_p H_{\boldsymbol{k}} \hat{\mathcal{T}}_p^{-1} = -H_{-\boldsymbol{k}}$.

3. Under chiral transformation, $\hat{\mathcal{C}} = I \otimes \sigma_z \otimes \sigma_z$, all of the link variables h_j and h_j' have odd chirality: $(\sigma_z \otimes \sigma_z) h_j (\sigma_z \otimes \sigma_z)^{-1} = -h_j$ and $(\sigma_z \otimes \sigma_z) h_j' (\sigma_z \otimes \sigma_z)^{-1} = -h_j'$, which leads to $\hat{\mathcal{C}} H_{\boldsymbol{k}} \hat{\mathcal{C}}^{-1} = -H_{\boldsymbol{k}}$.

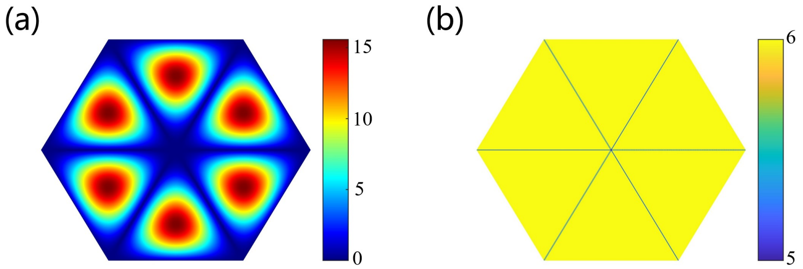

Supplementary Figure 3. (a) The determinant and (b) the rank of $\mathcal{Q}(\boldsymbol{k})$ are plotted as the function of lattice momentum $\boldsymbol{k}$ in the BZ.

Due to the link variables h_j and h_j' realizing single valley-flip of dipole up (C site) and dipole down (B site) excitons, respectively, the Hamiltonian is transformed into off-diagonal blocks only by selecting the basis $\hat{b}_{\boldsymbol{k}} = [\Psi^1_{\boldsymbol{k}}, \Psi^2_{\boldsymbol{k}}]^T$. The anti-diagonal form of the Hamiltonian Eq. S9 indicates the presence of chiral symmetry [13]. Here, $\Psi^1_{\boldsymbol{k}}$ resides in the valley pseudospin subspace spanned by $\{|K\rangle_B |K\rangle_C, |-K\rangle_B |-K\rangle_C\}$, while $\Psi^2_{\boldsymbol{k}}$ belongs to its complementary subspace spanned by $\{|K\rangle_B |-K\rangle_C, |-K\rangle_B |K\rangle_C\}$. The Hamiltonian Eq. S9 is then transformed as:

$$H_{\boldsymbol{k}} = t_f \begin{pmatrix} 0 & \mathcal{Q}(\boldsymbol{k}) \\ \mathcal{Q}^\dagger(\boldsymbol{k}) & 0 \end{pmatrix}$$

$$\mathcal{Q}(\boldsymbol{k}) = \begin{pmatrix} 0 & 1 & e^{i\frac{2\pi}{3}} & 0 & e^{i\boldsymbol{k}\cdot\boldsymbol{\delta}_1} & e^{-i\boldsymbol{k}\cdot\boldsymbol{\delta}_3 + i\frac{2\pi}{3}} \\ 1 & 0 & e^{-i\frac{2\pi}{3}} & e^{-i\boldsymbol{k}\cdot\boldsymbol{\delta}_1} & 0 & e^{i\boldsymbol{k}\cdot\boldsymbol{\delta}_2 - i\frac{2\pi}{3}} \\ e^{i\frac{2\pi}{3}} & e^{-i\frac{2\pi}{3}} & 0 & e^{i\boldsymbol{k}\cdot\boldsymbol{\delta}_3 + i\frac{2\pi}{3}} & e^{-i\boldsymbol{k}\cdot\boldsymbol{\delta}_2 - i\frac{2\pi}{3}} & 0 \\ 0 & e^{i\boldsymbol{k}\cdot\boldsymbol{\delta}_1} & e^{-i\boldsymbol{k}\cdot\boldsymbol{\delta}_3 - i\frac{2\pi}{3}} & 0 & 1 & e^{-i\frac{2\pi}{3}} \\ e^{-i\boldsymbol{k}\cdot\boldsymbol{\delta}_1} & 0 & e^{i\boldsymbol{k}\cdot\boldsymbol{\delta}_2 + i\frac{2\pi}{3}} & 1 & 0 & e^{i\frac{2\pi}{3}} \\ e^{i\boldsymbol{k}\cdot\boldsymbol{\delta}_3 - i\frac{2\pi}{3}} & e^{-i\boldsymbol{k}\cdot\boldsymbol{\delta}_2 + i\frac{2\pi}{3}} & 0 & e^{-i\frac{2\pi}{3}} & e^{i\frac{2\pi}{3}} & 0 \end{pmatrix} \tag{S11}$$

Here, $\mathcal{Q}(\boldsymbol{k})\Psi^2_{\boldsymbol{k}} = 0$ having non-zero solutions indicates the existence of zero mode, and it also ensures another zero mode $\mathcal{Q}^\dagger(\boldsymbol{k})\Psi^1_{\boldsymbol{k}} = 0$. The zero determinant of the off-diagonal matrix $\det \mathcal{Q}(\boldsymbol{k}) = 0$ guarantees the eigen-equation have non-zero solutions $\Psi^2_{\boldsymbol{k}}$. By the rank-nullity theorem, the number of the non-zero solutions is determined by $6 - \operatorname{rank} \mathcal{Q}(\boldsymbol{k})$. Due to $\operatorname{rank} \mathcal{Q}(\boldsymbol{k}) = \operatorname{rank} \mathcal{Q}^\dagger(\boldsymbol{k})$, the total number of zero modes is given by $12 - 2 \operatorname{rank} \mathcal{Q}(\boldsymbol{k})$. In our work, the $|\det \mathcal{Q}(\boldsymbol{k})|$ and $\operatorname{rank} \mathcal{Q}(\boldsymbol{k})$ are calculated in the BZ as shown in Supplementary Figure 3a and Supplementary

Figure 3b, respectively. Along high-symmetry lines, det $\mathcal{Q}(\boldsymbol{k}) = 0$ and rank $\mathcal{Q}(\boldsymbol{k}) = 5$ indicate that there are twofold degenerate nodal-ring zero modes.

[1] H. Yu, G.-B. Liu, J. Tang, X. Xu, and W. Yao, Moiré excitons: From programmable quantum emitter arrays to spin-orbit–coupled artificial lattices, Science Advances **3**, e1701696 (2017).

[2] H. Yu, Y. Wang, Q. Tong, X. Xu, and W. Yao, Anomalous light cones and valley optical selection rules of interlayer excitons in twisted heterobilayers, Phys. Rev. Lett. **115**, 187002 (2015).

[3] H. Yu and W. Yao, Luminescence anomaly of dipolar valley excitons in homobilayer semiconductor moiré superlattices, Phys. Rev. X **11**, 021042 (2021).

[4] Y. Wang, Z. Wang, W. Yao, G.-B. Liu, and H. Yu, Interlayer coupling in commensurate and incommensurate bilayer structures of transition-metal dichalcogenides, Phys. Rev. B **95**, 115429 (2017).

[5] H. Zheng, C. Li, H. Yu, and W. Yao, Förster valley-orbit coupling and topological lattice of hybrid moiré excitons (2024), arXiv:2410.03443 [cond-mat.mes-hall].

[6] H. Yu, G.-B. Liu, J. Tang, X. Xu, and W. Yao, Moiré excitons: From programmable quantum emitter arrays to spin-orbit–coupled artificial lattices, Science Advances **3**, e1701696 (2017).

[7] F. Wu, T. Lovorn, E. Tutuc, I. Martin, and A. MacDonald, Topological Insulators in Twisted Transition Metal Dichalcogenide Homobilayers, Physical Review Letters **122**, 086402 (2019).

[8] N. Marzari, A. A. Mostofi, J. R. Yates, I. Souza, and D. Vanderbilt, Maximally localized wannier functions: Theory and applications, Rev. Mod. Phys. **84**, 1419 (2012).

[9] P. Cudazzo, I. V. Tokatly, and A. Rubio, Dielectric screening in two-dimensional insulators: Implications for excitonic and impurity states in graphane, Phys. Rev. B **84**, 085406 (2011).

[10] D. A. Ruiz-Tijerina, I. Soltero, and F. Mireles, Theory of moiré localized excitons in transition metal dichalcogenide heterobilayers, Physical Review B **102**, 195403 (2020).

[11] J. Kutrowska-Girzycka, E. Zieba-Ostój, D. Biegańska, M. Florian, A. Steinhoff, E. Rogowicz, P. Mrowiński, K. Watanabe, T. Taniguchi, C. Gies, S. Tongay, C. Schneider, and M. Syperek, Exploring the effect of dielectric screening on neutral and charged-exciton properties in monolayer and bilayer MoTe2, Applied Physics Reviews **9**, 041410 (2022).

[12] W. Krauth, *Statistical mechanics: algorithms and computations*, Vol. 13 (OUP Oxford, 2006).

[13] J. X. Dai and Y. X. Zhao, Topological classification for chiral symmetry with non-equal sublattices (2024), arXiv:2405.16001 [cond-mat].
